# Theranostic Nanoparticles Based on a Silicon Dioxide Core, a Mercapto Spacer, a Cardioprotector, and a Fluorescent Dye

**DOI:** 10.3390/ijms27114844

**Published:** 2026-05-27

**Authors:** Dmitrii V. Korolev, Galina A. Shulmeyster, Maria A. Baybakova, Ilia E. Anufriev, Michael M. Galagudza

**Affiliations:** 1 Institute of Experimental Medicine, Almazov National Medical Research Centre, 15B Parkhomenko Ave., 197341 Saint-Petersburg, Russia; g.schulmeister@yandex.ru (G.A.S.); knikee@mail.ru (M.A.B.); ilya_anufriev_00@mail.ru (I.E.A.); galagudza@almazovcentre.ru (M.M.G.); 2 Laboratory of Biophysics of Blood Circulation, Pavlov First Saint-Petersburg State Medical University, St. Lev Tolstoy 6/8, 197022 Saint-Petersburg, Russia; 3 Department of Micro-and Nanoelectronics, Saint-Petersburg Electrotechnical University “LETI”, St. Prof. Popov, 5f, 197376 Saint-Petersburg, Russia

**Keywords:** theranostics, nanoparticles, fluorophore, cardioprotector, mercapto spacer

## Abstract

A method for synthesizing theranostic nanoparticles (NPs) based on a silica core, a mercapto spacer, a cardioprotector, and a fluorescent dye has been developed. The total amount of grafted mercapto groups was 0.079 mmol/g. The amount of accessible mercapto groups on the surface of the synthesized particles, calculated using the Kunkel, Buckley, and Gorin method, was 0.025 mmol/g. A total of 0.031 mmol/g of adenosine and 0.0087 mmol/g of indocyanine green are grafted onto the mercapto spacer. Both substances are presumably attached via hydrogen bonding to the modified silica nanoparticle in a ratio of 60/40% for adenosine and indocyanine green, respectively. The resulting nanoparticles exhibit no hemolytic activity. Intensive adenosine release occurs within 90 min and continues for up to 24 h. Based on biodistribution, significant accumulation of the nanoparticles occurs in the liver.

## 1. Introduction

Nanoparticles (NPs) enable the delivery of a wide range of drugs, including anticancer drugs, analgesics, Alzheimer’s disease drugs, cardiovascular drugs, protease inhibitors, and a number of macromolecules, to the brain after intravenous injection in laboratory animals [1]. Some of them are already used in clinical practice to redesign contrast agents and drugs and are called “smart” particles [2]. For example, peptide-based NPs, developed using new principles of molecular engineering, demonstrate enormous potential for overcoming many of the obstacles faced by NPs made from more traditional materials during drug delivery [3]. Gold nanoparticles have low toxicity, penetrate brain endothelium in vitro and in vivo, and rapidly move through brain parenchyma [4]. Within minutes of infusion, nanoparticles can be detected in neurons and glial cells. In addition to the well-established strategy of creating prodrugs, chemical conjugation of transporter substrates with nanoparticles of various chemical compositions has recently been used to improve their targeting and absorption [5]. For example, nanomedicine has opened up opportunities for the development of new platforms that provide targeted drug delivery during pregnancy, significantly reducing the risk of complications [6].

For many years, injectable polymeric NPs have been developed for the delivery of therapeutic agents to tumors. The NP surface has often been modified with various moieties and/or ligands to impart a latent effect and/or induce specific cellular interactions, which are known to significantly influence the fate and efficacy of these NPs in vivo [7]. It is well known that some NPs, such as silver, zinc oxide, copper, iron, etc., exhibit significant antimicrobial activity because they release metal ions that subsequently generate reactive oxygen species (ROS), demonstrating potential as a better alternative to antibiotics and other antimicrobial drugs [8]. DNA–nanoparticle conjugates [9] exist, which are hybrid nanoobjects combining various types of DNA molecules and inorganic nanoparticles with a typical DNA shell architecture around an inorganic core. This combination provides the particles with the unique properties of DNA, addressability and recognition, but at the same time makes it possible to utilize the properties of the inorganic core of the particle. For example, a recombinant HALO-GFP hybrid protein was developed and isolated, which was used to modify gold nanoparticles [10]. Dispersing agents [11] have been optimized to achieve precise control over the stability, size and functional properties of nanoparticles by independently taking into account the influence of anchoring groups and attached sterically repulsive polymer ligands. Of interest are nanoparticles coated with various shells, such as poly(lactic-co-glycolic acid) (PLGA) [12] or cell membranes [13], ensuring the non-toxicity of any nanomaterials. An integrated approach [14] has been developed for site-selective conjugation between aminated silicon nanoparticles (SiNPs) and the only available thiol in human serum albumin (HSA). A new method for creating a zwitterionic layer based on amino acids on silicon nanoparticles functionalized with (3-aminopropyl)triethoxysilane (APTES) has been developed [15]. Such nanoparticles do not become coated with proteins and do not form a protein corona.

Pre-modification using 3-mercaptopropyltrimethoxysilane in xylene was used to immobilize ethyleneimine on silica gel [16]. In another study, MSM-41 grade silica was dispersed in dry toluene, mixed with MPTS, and refluxed for 12 h under a nitrogen atmosphere [17]. The thiol-modified MSM-41 silica was washed with ethanol and lyophilized.

Covalent bonding through the mercapto groups (-SH) of trypsin in the channels of synthesized mesoporous silica microspheres (CSMS) was demonstrated [18]. For modification with the -SH group, CSMS particles were dispersed in toluene, followed by the addition of MPTS to the solution. The mixture was then refluxed at 110 °C for 12 h. The modified CSMS particles were recovered by centrifugation, washed three times with ethanol, and dried under vacuum at room temperature.

The production of a hybrid monolithic PImC8-silica column was described [19]. MPTS was used as a spacer. Here, as in our case, it was grafted to the surface.

Mesoporous graphene aerogel was synthesized using MPTS [20]. MPTS was mixed with a hydrochloric acid solution with a pH of 4 to 5. A dispersion of graphene oxide (GO) was then mixed with the resulting MPTS/HCl solution via ultrasonic dispersion. Hydrothermal synthesis was then performed. The mixture was heated to 200 °C and maintained for 8 h in a Teflon reactor to complete the hydrolysis, functionalization, and self-assembly process.

A unique and personalized nanocarrier for controlled and targeted delivery of anticancer drugs and real-time bioimaging, created by combining a disulfide-conjugated carbon dot, responsive to redox processes and enzymes, with mesoporous silica nanoparticles, is described in [21]. The carbon dot with the ability to control and target was obtained through a polymerization reaction using citric acid and hyaluronic acid as raw materials.

The effect of functionalization of -SH groups on the stability, solubility, and plasmonic properties of gold nanoparticles with the general formula Au_18_(SH)_14_ in an aqueous solvent was studied [22]. Various modifiers were used: 1,1-mercaptoethyl alcohol, s-cysteamine, thioglycolic acid, and beta-mercaptoethanol. An increase in hardness and, consequently, chemical stability was observed for the functionalized nanoparticles compared to the pure structure. Based on the obtained data from quantum mechanical calculations and molecular dynamics simulations, it has been concluded that the particle with 1,1-mercaptoethyl alcohol is the best ligand for increasing the solubility, stability, and plasmonic functions of gold-based structures.

Summary data on the synthesis parameters of the -SH spacer are presented in Table 1.

The literature presents data on the reduction in nanoparticle toxicity by creating a shell made of MPTS. For example, carbon quantum dots with such a shell are completely non-toxic [23], the toxicity of CuS/ZnS nanocrystals [24] and even porous silicon dioxide MCM-40 loaded with cisplatin [25] is reduced.

The aim of this study was to develop a method for synthesizing theranostic nanoparticles based on a silica core, a mercapto spacer, a cardioprotective agent adenosine (ADN), and a fluorescent dye, and to characterize them.

## 2. Results and Discussion

### 2.1. Measurement of Physical and Chemical Properties

The hydrodynamic size of silica nanoparticles negligibly increases during the reaction with MPTS. However, the reaction with MPTS in benzene produces a large number of agglomerates, which likely indicates some polymer formation from the modifier. The low value of the polydispersity index allows us to state that the size distribution is close to monodisperse. The zeta potential increases in magnitude with the introduction of the –SH group from −32.02 ± 2.07 to −40.72 ± 1.12 mV, making the colloidal solution more stable (Table 2).

### 2.2. The Amount of –SH Spacer by Argentometry

**Figure 1:**
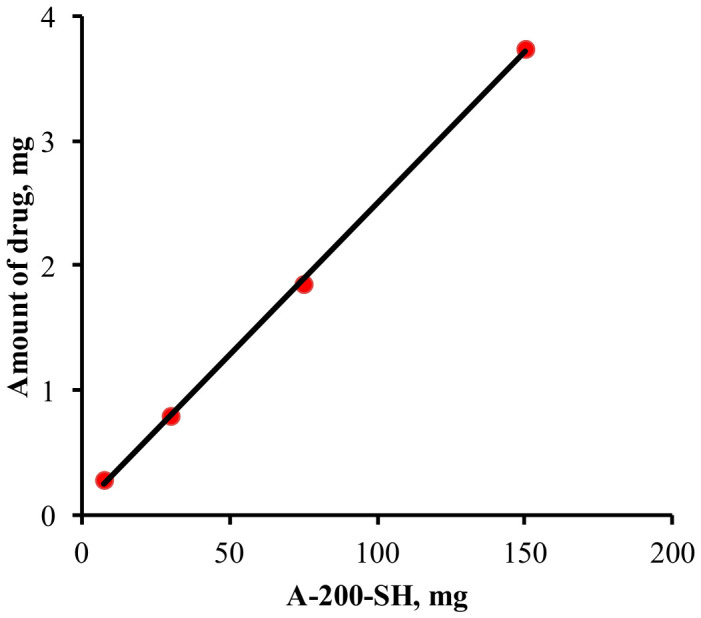
Quantitative graph of the total –SH group content.

Figure 1 shows the results of titration to determine the total amount of –SH groups on the surface of aerosil by argentometry.

Approximating the graphs through the origin, we obtain the total mercapto group content on the surface of the synthesized particles. The total –SH group content on the surface of the synthesized particles was 0.079 ± 0.001 mmol/g.

The amount of accessible –SH groups on the surface of the synthesized particles, calculated using the Kunkel, Buckley, and Gorin method, was 0.025 ± 0.001 mmol/g.

The composition of A-200-SH nanoparticles was studied using X-ray fluorescence analysis.

**Figure 2:**
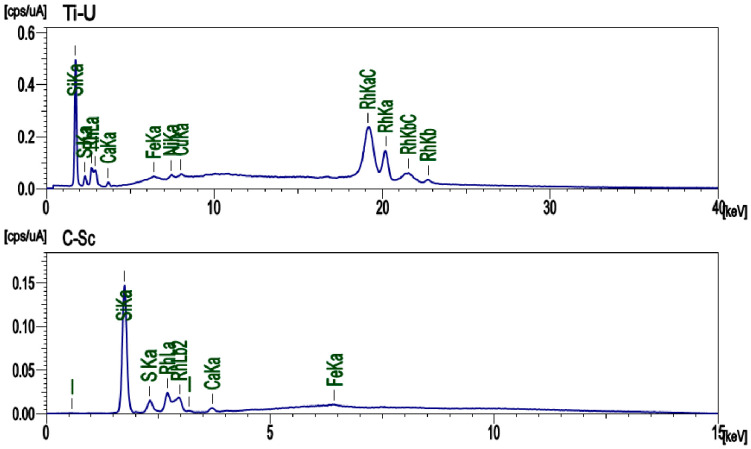
X-ray diffraction pattern of sample A-200-SH.

The results are shown in Table 3; the X-ray diffraction pattern is shown in Figure 2.

Considering that the X-ray diffraction method does not allow one to determine the amounts of oxygen and carbon, and that the A-200 nanoparticle consists of silica and sulfur is immobilized using a propyl radical, one can theoretically calculate the mass content of sulfur in terms of SiO_2_ (1.586% by weight) and, taking into account the mass of the propyl radical, it is 0.590% by weight or 0.18 mmol/g. Thus, the difference from the total content of –SH groups determined by argentometry (including the hydrogen atom) is 2.278 times. The maximum measurement error in this case can be considered the instrumental deviation for sulfur—0.031%.

### 2.3. The Amount of Grafted ICG and Adenosine

Using calibration curves and obtaining optical density values after desorption, we find that 0.031 mmol/g adenosine and 0.0087 mmol/g ICG were grafted onto the mercapto spacer. Thus, both substances bind via hydrogen bonding with the modified silica nanoparticle in a mass ratio of 60% adenosine and 40% indocyanine green, respectively. Moreover, the larger amount of sorbed adenosine than the available -SH groups can be explained by the particle formation of dimers or physical adsorption on the surface of nanoparticles.

### 2.4. FTIR Spectra

**Figure 3:**
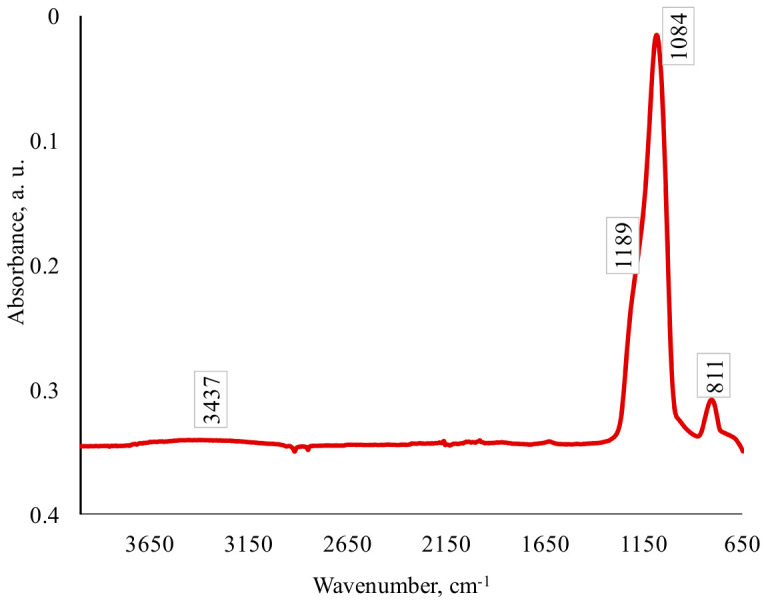
IR spectrum of SiO_2_ nanoparticles treated with MPTS.

**Figure 4:**
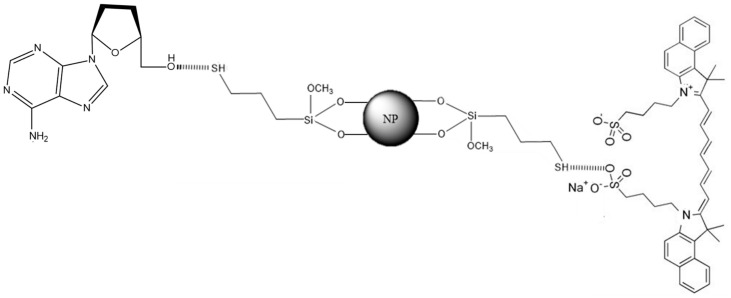
Theranostic nanoparticle based on the –SH spacer.

According to [26], –SH groups have very weakly defined lines in the FTIR spectra. Therefore, the presence of a modifier on the surface of nanoparticles can only be judged by indirect signs, that is, by the presence of other bond lines of this modifier. Thus, according to [27,28], Si-O-Si (1084 cm^−1^) and Si-OH bond lines (Figure 3) were detected. This indicates that the modification of SiO_2_ nanoparticles does indeed proceed according to the scheme shown in Figure 4, and a Si-O-Si bond is formed, but some of the –OH groups are not reactive. Vibrations of the Si-C bond of the modifier are also present.

**Figure 5:**
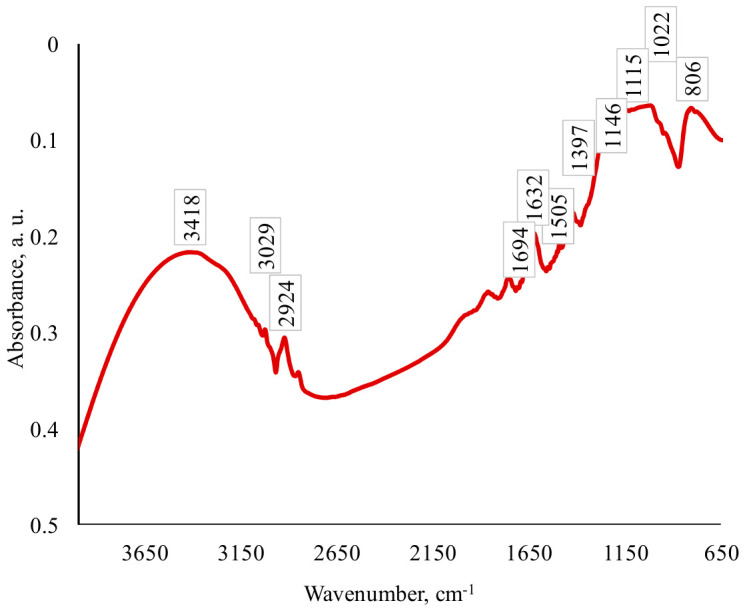
IR spectrum of the theranostic nanoparticle.

Figure 5 shows the IR spectrum of a theranostic nanoparticle comprising silica modified with a –SH spacer and the fluorescent dye indocyanine green and the drug adenosine grafted onto its surface. As can be seen from Figure 5, characteristic peaks corresponding to the following compounds were detected: indocyanine green—1111 cm^−1^ S=O vibrations in SO_3_-, 1383 cm^−1^ C-N vibrations, 1501 cm^−1^ C=C vibrations; adenosine—1020 cm^−1^ vibrations of the C-O and C-O-C valence bonds in the sucrose ring of ribose, 1631 cm^−1^ deformation vibrations of -NH_2_, 1689 cm^−1^ vibrations of C=O, 2926 cm^−1^ vibrations of C-H vibrations of alkyl bonds in the sucrose ring.

### 2.5. Thermogravimetric Analysis

**Figure 6:**
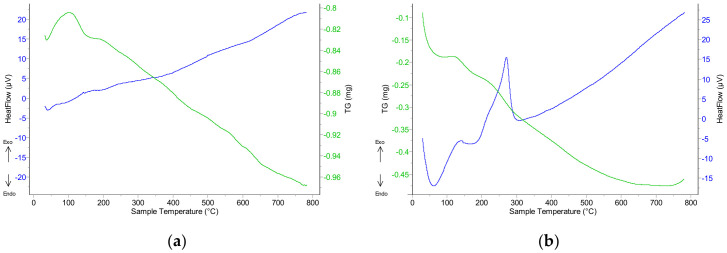
Thermograms of the sample: (**a**) in an inert environment; (**b**) in an oxygen environment.

The thermograms of the sample are shown in Figure 6. In an inert atmosphere (Figure 6a), water evaporates from the sample in the temperature range of 100–150 °C, accompanied by weight loss. This is followed by monotonic decomposition of the sample throughout the studied temperature range. In an oxygen environment (Figure 6b), water removal occurs earlier at 20–120 °C. This is likely due to the different thermal conductivities of the gases. Sample decomposition occurs in the temperature range of 200–300 °C and is accompanied by a strong exothermic effect. The weight loss in an inert gas is insignificant, 0.16 mg, with a sample weight of 5.90 mg (2.7%), while in oxygen it is 0.45 mg with a sample weight of 5.20 mg (8.6%).

### 2.6. Transmission Electron Microscopy

**Figure 7:**
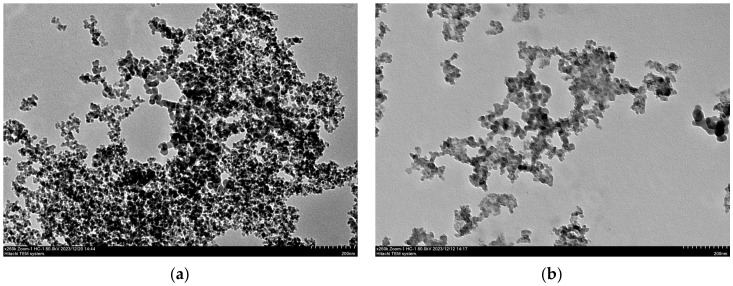
Electron micrographs of samples: (**a**) before modification; (**b**) after modification.

Micrographs of unmodified samples (Figure 7a) show loose particles measuring 8–10 nm. Modification results in the formation of a shell and agglomerates of varying sizes (Figure 7b).

### 2.7. Study of Natural Biodistribution

The results of animal respiratory rate measurements are presented in Table 4. It remained virtually unchanged throughout the experiment, which indirectly indicates the absence of a toxic effect, nanoparticle aggregation, and thrombosis.

**Figure 8:**
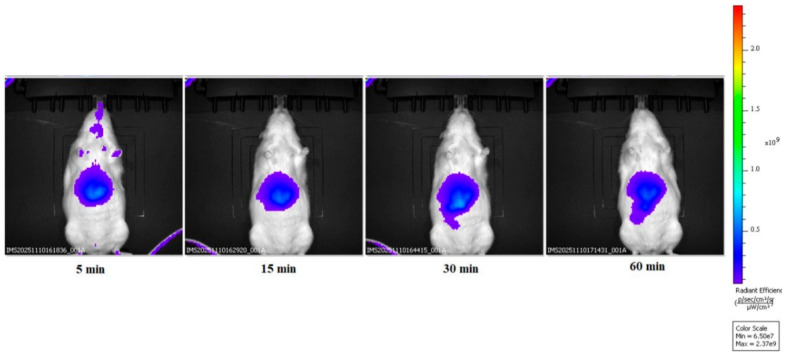
Biodistribution of NPs within rats.

Dynamic signal recording (Figure 8) shows the distribution of nanoparticles over time, with the maximum accumulation of particles in the liver region occurring at 15 min and remaining virtually unchanged after that.

**Figure 9:**
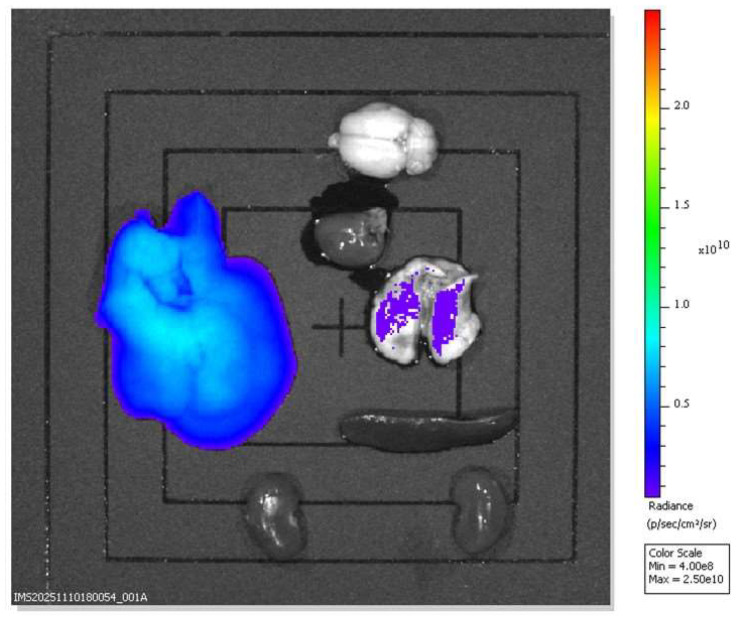
Biodistribution of NPs within organs.

Ex vivo studies were conducted on rat organs 60 min after intravenous administration of nanoparticles. Organs of interest, such as the brain, heart, lungs, kidneys, spleen, and liver, were analyzed using the same fluorescence imaging settings as in the in vivo study, and ROIs were marked along the organ contours to assess fluorescence intensity (Figure 9). The highest accumulation of NPs was recorded in the liver (Table 5), suggesting a pathway for their bioelimination. A small amount of NPs was also detected in the lungs, suggesting that they accumulate in the area of small vessels.

### 2.8. Hemolytic Activity

According to the results of Table 6, the percentage of hemolysis does not reach even 5%, which indicates the safety and non-toxicity of used theranostic nanoparticles.

### 2.9. Release Profile of Active Ingredients

**Figure 10:**
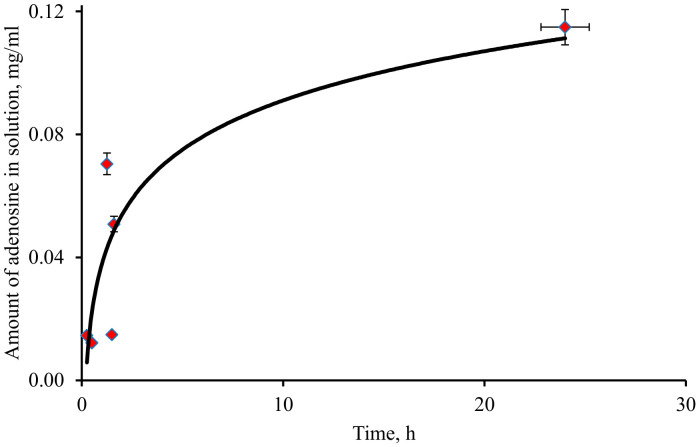
Release profile of adenosine from the NPs-SH-ADN.

There is no noticeable release of adenosine during initial 90 min post-incubation. Significant release of adenosine occurs only after 90 min and continues for 24 h (Figure 10). This indicates good chemisorption, allowing for a prolonged effect.

### 2.10. Effect of a Theranostic Drug on Myocardial Infarction Size in Rats Using a Model of Coronary Occlusion Myocardial Infarction

In the control group, the infarction size was 52.8 ± 6.3%, and in the experimental group it was 34.3 ± 10.3% (*p* < 0.05).

### 2.11. The Possibility of Visualizing the Myocardial Damage Zone Using Fluorescence Tomography After the Introduction of Theranostic Nanoparticles

The area of positively stained areas was 38.4 ± 9.7%, which is not significantly different from the infarction size obtained by the standard histochemical method.

## 3. Materials and Methods

### 3.1. Synthesis

**Figure 11:**
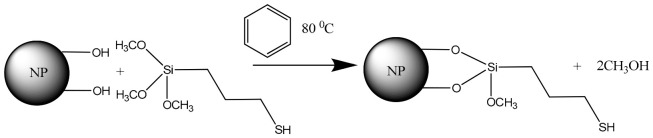
Formation of –SH groups on the surface of the nanoparticle shell.

By boiling silica nanoparticles in benzene in the presence of the MPTS modifier, a shell with a functional -SH group was formed. The reaction scheme is shown in Figure 11. Many textbooks, for example [29], indicate that the modification of the surface of trialkoxysilanes A-200 by organic silanes occurs through one Si-O-Si bond. However, many textbooks, for example [30], indicate that the modification of the surface of A-200 by organic silanes occurs through two Si-O-Si bonds.

The modification was carried out as follows. A 100 mL round-bottomed, three-necked flask was charged with 1 g of Aerosil A-200 (Degussa, Frankfurt am Main, Germany), 23.75 mL of benzene (Vekton, Saint-Petersburg, Russia), and 1.25 mL of MPMS (Sigma-Aldrich, Burlington, MA, USA). The mixture was refluxed for two hours at the solvent’s boiling point. After completion of the synthesis, the particles were washed three times with cyclohexane to remove excess reagents and then freeze-dried.

Theranostic nanoparticles were prepared by simultaneously adding the fluorescent dye ICG and adenosine (Sigma-Aldrich, Shanghai, China). For this purpose, a solution of ICG and adenosine was prepared in a ratio of 2 mg and 9 mg, respectively, and added to 50 mg of dry silica nanoparticles with a mercapto spacer. The reaction mixture was stirred on an orbital shaker at 300 min^−1^ for 2 h. Excess reagents were then washed off by centrifugation.

**Figure 12:**
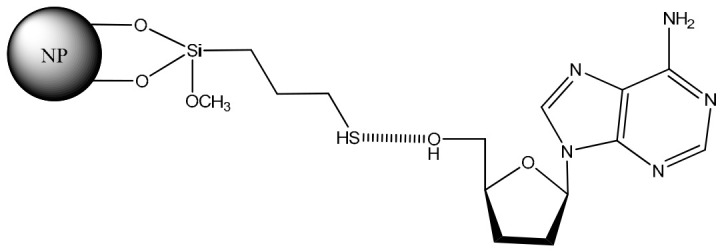
Interaction of –SH and the hydroxyl group of adenosine.

**Figure 13:**
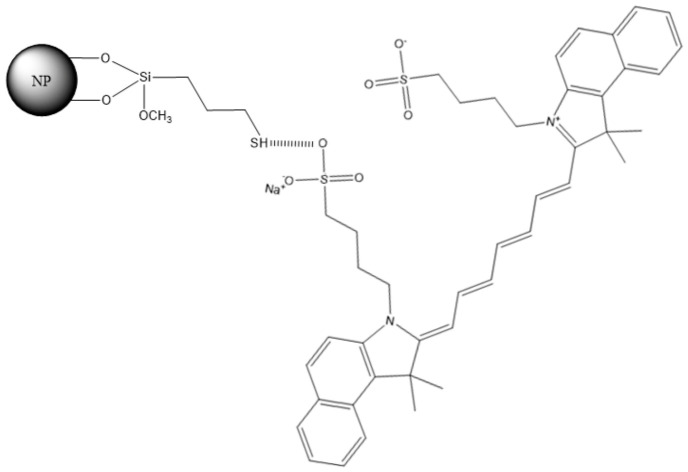
Interaction of –SH and oxygen from the sulfo group of ICG.

The hydroxyl group of adenosine (-OH) can form hydrogen bonds with the -SH group (Figure 12). Indocyanine green also appears to interact with the -SH group by forming a hydrogen bond through the oxygen atom in the sulfo group (-SO_3_) (Figure 13).

### 3.2. Determination of –SH Group Quantity

The total amount of –SH groups was determined by argentometry, which is an indirect titration method. The test substance was dissolved in 15 mL of water. A total of 1 mL of 0.04% (0.4 mg/mL) bromothymol blue was added and mixed. Then 5 mL of 0.1 M silver nitrate was added and mixed. Next, titration was started with a 0.1 M sodium hydroxide solution, diluted 10-fold for convenience, from the supernatant solution turning yellow until a persistent green color was achieved (pH = 7.0). One ml of 0.1 M sodium hydroxide solution corresponds to 11.42 mg of the drug [31]. To determine the amount of mercapto groups available for chemisorption on the surface of aerosil, the Kunkel, Buckley, and Gorin method was used. Silver dithizonate was prepared. In a 100 mL volumetric flask, 0.01 g of dithizone was added and carbon tetrachloride was poured in. The dye was dissolved as much as possible, resulting in a dark green solution, while the aqueous solution of dithizone was bright pink [32]. Next, 0.5 g of silver nitrate was dissolved in 50 mL of water, and these two solutions were placed in a 250 mL conical flask. Nothing happened during mixing, since these are two immiscible liquids. Then 2.5 mL of 5 M H_2_SO_4_ was added and the mixture was stirred vigorously for 5–10 min. A layer of red-orange oily liquid was obtained at the bottom and water on top, while undissolved and unreacted substances accumulated at the interface. Next, silver dithizonate was washed with water using a separatory funnel: the heavy red-orange liquid (silver dithizonate) was poured off, the residues were removed, clean water was poured into the silver dithizonate, and this was repeated 3 times. The resulting silver dithizonate, with a volume of less than 100 mL, was stored in the refrigerator. For measurements, the resulting silver dithizonate was diluted 5-fold, yielding a maximum optical density relative to CCl_4_ close to unity using a spectrophotometer with quartz cuvettes at 460 nm.

### 3.3. Amount of Grafted ICG and Adenosine

A calibration curve was plotted for the optical density versus ICG concentration relative to distilled water at a wavelength of 700 nm. The ICG concentrations were 0.031, 0.016, 0.008, and 0.004 mg/mL. To determine the amount of adenosine, a calibration curve was constructed. A 1 mg/mL adenosine solution was successively diluted with 0.1 N NaOH and distilled water by 16, 32, 64, and 128 times. To ensure adenosine hydrolysis, the optical density of the solution was measured in neutral and alkaline media. The study was conducted at a wavelength of λ = 260 nm. A calibration curve was constructed for both neutral and alkaline media.

To determine immobilized ICG and adenosine, desorption was performed by adding 10 mL of 0.1 N NaOH solution to the washed precipitate and leaving it on a shaker for 15 min. The particles were then precipitated by centrifugation, and the supernatant was used for spectrophotometric analysis. It was diluted to ensure that the maximum relative optical density was no more than 1 at 700 nm for ICG and at 260 nm for adenosine. The optical density data were plotted against calibration curves, and the amounts of the substances were calculated.

### 3.4. Physicochemical Properties

The structure of the obtained products was confirmed by solid-state IR spectrometry. Spectra were obtained in the wavenumber range from 7500 to 300 cm^−1^ using an InfraLUM FT-08 instrument (Lumex, Saint-Petersburg, Russia) using the following parameters: apodization—Happ–Genzel, scan count—60, resolution—4.0 cm^−1^. The sample was prepared with dry KBr.

Tablet preparation. A weighed portion of the analyzed sample was thoroughly ground with crystalline potassium bromide in an agate mortar and pestle. The sample/KBr ratio was 5/95 mg. The resulting mixture was placed in a special mold and pressed using a hydraulic press with a maximum force of 10 tons. The resulting sample was pressed into a tablet with a diameter of 13 mm. The absorption spectra of the samples were analyzed using a Unico 2802S dual-beam spectrophotometer (Unico Sys, Arnold, MO, USA).

The hydrodynamic diameter of the nanomaterials and the polydispersity index in aqueous solutions were determined using dynamic light scattering (DLS) (Zeta Sizer Ultra, Malvern Instruments, Malvern, UK). The following instrument parameters were used: correlation function accumulation time was 120 s, size distribution was measured in triplicate, sample temperature was 25 °C, and the general purpose analysis method was used. Additional settings were set to default values. Dedusting of the colloidal solution was not performed. The mean hydrodynamic size was determined by the Z-average value, which was calculated from the raw size distribution intensity data.

The composition of A200-SH was studied using an energy-dispersive X-ray fluorescence spectrometer (EDX 800 HS, Shimadzu, Kyoto, Japan), a tube with an Rh anode, 5–50 kV, current 1–1000 μA.

Thermogravimetric analysis was performed using a SETSYS-1750 CS Evol (Setaram, Caluire, France) in the temperature range of 20–800 °C in both oxygen and argon atmospheres.

The morphology of the samples was examined using an transmission electron microscope (TEM) (HT7800 Series, Hitachi, Hitachinaka City, Japan). Nickel Grids Formvar Support Films 200 Mesh (Zongjingkei Technology, Shenzhen, Guangdong Province, China) were used as substrates.

### 3.5. Biodistribution Analysis

In vivo and ex vivo fluorescence analysis was performed using a non-invasive, highly sensitive bioimaging system (IVIS Lumina LT Series III, PerkinElmer, Waltham, MA, USA). The study was conducted on Wistar rats (Pushchino Laboratory Animal Facility). This study was conducted in strict accordance with the National Institutes of Health’s Guide for the Care and Use of Laboratory Animals. The protocol was approved by the Institutional Animal Care and Use Committee (IACUC). Animal studies were conducted with the approval of the local ethics committee of the Almazov National Medical Research Center (protocol 21-05PZ#V2 dated 23 March 2021).

Imaging parameters were adjusted based on the indocyanine green dye and corresponded to wavelengths of 745 nm for absorption and 835 nm for emission. The following settings were used for fluorescence imaging: binning—8, exposure—Auto.

Fluorescence image processing was performed using the built-in Living Image 4.7.4 software (PerkinElmer, Waltham, MA, USA). Fluorescence signals were read from the color maps of the acquired images by selecting the ROI (region of interest). ROIs encompassing accumulation areas and organs were drawn manually, after which the software calculated the total irradiance for each ROI. Thus, the total irradiance is proportional to both the organ surface area in the image (ROI area) and the fluorescence signal of each pixel within the ROI.

### 3.6. Assessment of Hemolytic Activity

The toxicity of the resulting nanoparticles was tested by studying their hemolytic activity on whole blood from 3 donors. For analysis, the resulting nanoparticles were mixed with whole blood and placed in an incubator at 37 °C. Control points were set at 1, 2, 4, and 24 h, and the samples were centrifuged for 20 min at 2000 rpm. A total of 200 μL of the supernatant was mixed with 5.8 mL of saline and measured spectrophotometrically at 415 nm against a blank saline sample. The percentage of hemolysis was calculated using the formula:

*Q* = (*Dtotal* − *Dnc*)/*Dpc* × 100%

where *Dtotal* is the optical density of the test sample, and *Dnc* and *Dpc* are the optical density of the negative and positive controls, respectively.

### 3.7. Release Profile of Active Substances

A 50 mL NP sample was placed in a dialysis bag with a pore diameter of 3.5 kDa, a width of 33 mm, and a length of 120 mm. The edges of the bag were secured with standard clamps. The collected bag was placed in a 1000 mL glass beaker, into which 500 mL of phosphate-buffered saline (PBS) with a pH of 7.4 was added. The sample was stirred on a magnetic stirrer at a speed of 150 s^−1^. Aliquots of 2 mL were periodically collected. This volume was compensated for with freshly prepared PBS buffer. The collected aliquots were analyzed spectrophotometrically.

### 3.8. Evaluation of the Effect of a Theranostic Drug on Myocardial Infarction Size in Rats Using a Model of Myocardial Infarction

Experiments were conducted on male Wistar rats weighing 200–250 g. The animals were anesthetized with a single intraperitoneal injection of chloral hydrate (Acros Organics, Pittsburgh, PA, USA) at a dose of 450 mg/kg. Artificial lung ventilation was performed through tracheal intubation with preliminary treatment of the larynx with a 2% lidocaine solution (respiratory rate—60/min, tidal volume—3 mL/100 g body weight). Access to the heart was established through the fourth intercostal space with a preliminary L-shaped skin incision from the upper edge of the body of the sternum to the xiphoid process along the midline and further along the 7th rib to the midaxillary line and separation of the pectoral muscles. A ligature was placed under the left coronary artery and an occluder was formed, after which myocardial ischemia lasting 30 min and reperfusion lasting 90 min were simulated. Animals were randomly divided into 2 groups: (1) control (n = 5) and (2) experimental (n = 5). Animals in the control group were intravenously administered adenosine in saline in a volume of 1 mL and a dose of 300 μg/kg/min for 10 min (from the 25th minute of ischemia to the 5th minute of reperfusion), and animals in the experimental group were administered theranostic nanoparticles in a dose equivalent to free adenosine in a volume of 1 mL of saline. After completion of reperfusion, the animals were euthanized by anesthesia overdose and the hearts were removed to assess the infarct size. The sizes of the risk zone and infarction zone were assessed using a double-staining technique with Evans blue and triphenyltetrazolium chloride. After reperfusion was completed, the ligature around the coronary artery was tightened again, and 0.5 mL of a 5% Evans blue solution was administered intravenously. After visualizing the boundary between the perfused and ischemic sections, the heart was quickly removed and cut transversely into four slices of equal thickness (2.5 mm). Images of the basal surfaces of the four slices were photographed with an Olympus 2020 digital camera (Olympus, Tokyo, Japan) connected to the microscope’s photomicrograph to subsequently determine the area of the anatomical risk zone (Evans-negative areas) and non-ischemic myocardium (Evans-positive areas). Area calculations were performed on a computer. The total risk zone volume for a given heart was calculated by multiplying the area of the Evans-negative area of each slice by its thickness and summing the values obtained for the four slices. The heart slices were then placed in a 1% TTC solution for 15 min at an incubation temperature of 37 °C. After incubation with TTC, the basal surfaces of the slices were re-photographed, and the infarct zone areas (TTC-negative areas within the risk zone) were calculated using a computer. The total infarct zone volume for a given heart was then calculated using the same method as for the risk zone. Data on the sizes of the risk and infarct zones were presented as the ratio of the risk zone volume to the total heart volume, as well as the ratio of the infarct zone volume to the risk zone volume (as a percentage).

## 4. Conclusions

Theranostic nanoparticles with a silica core grafted with the cardioprotector adenosine and the fluorescent dye indocyanine green were synthesized. The particles were indirectly characterized using IR spectroscopy. The total amount of –SH groups on the surface of the synthesized particles was 0.079 mmol/g. The amount of accessible –SH groups on the surface of the synthesized particles, calculated using the Kunkel, Buckley, and Gorin method, was 0.025 mmol/g. It was shown that 0.031 mmol/g of adenosine and 0.0087 mmol/g of ICG are grafted onto the –SH spacer. The resulting nanoparticles exhibit no hemolytic activity, indicating good biocompatibility. Intense adenosine release occurs within 90 min and continues for up to 24 h, prolonging the drug’s action. Biodistribution data revealed significant accumulation of nanoparticles in the liver, suggesting a metabolizing pathway.

## Figures and Tables

**Table 1:** Summary data on the synthesis of the -SH spacer.

Solvent	Synthesis Temperature, °C	Synthesis Time	Reference
Xylene (toluene)	139 (110.6)	72 h	[16]
Toluene	110.6	12 h	[17]
Toluene	110	12 h	[18]
PEG + acetic acid	55 (+US in 0 °C)	12 h	[19]
HCl pH 4–5	200 (+US)	8 h	[20]
Citric and hyaluronic acid solutions, ethylenediamine	120 (+US)	3 h	[22]

Note: US—ultrasonic treatment.

**Table 2:** Physicochemical properties of the A-200-SH.

Sample	Hydrodynamic Size, nm	Polydispersity Index	Zeta Potential, mV
A-200-SH	150.6 ± 4.7	0.17 ± 0.06	−33.04 ± 1.02
A-200-SH-ADN	171.8 ± 4.7	0.15 ± 0.01	−40.72 ± 1.12
A-200-SH-ADN-ICG	320.1 ± 4.8	0.22 ± 0.09	−32.02 ± 2.07

**Table 3:** Composition of A-200-SH nanoparticles based on X-ray fluorescence analysis.

Element	Si	S	Ca	Ni	Cu
Content, %	96.004	3.621	0.298	0.02	0.015
Sigma, %	0.231	0.031	0.005	0.001	0.001

**Table 4:** Animal respiratory rate upon drug administration.

Sample	Before Infusion	After Infusion	After 30 min	After 60 min
A200-SH-AND-ICG	54	51	44	47

**Table 5:** Numerical values of organ fluorescence after nanoparticle administration.

Organ	Average Radiance Efficiency, p/sec/sm^2^/sr × 10^8^
Liver	27.61
Brain	2.03
Heart	2.70
Lung	4.96
Spleen	4.87
Kidneys	5.23

**Table 6:** Results of the study of hemolytic activity of the studied samples.

Sample	Time, h	Hemolysis, %		
		Donor 1	Donor 2	Donor 3
A-200-SH-ADN	1	2.74	1.29	1.90
	2	1.82	0.71	1.01
	4	1.27	1.58	0.96
	24	0.60	0.79	1.04
A-200-SH-ADN-ICG	1	2.83	1.07	2.34
	2	1.62	1.11	1.06
	4	1.11	1.54	0.70
	24	0.89	1.51	0.80

## Data Availability

The original contributions presented in this study are included in the article. Further inquiries can be directed to the corresponding author.

## Abbreviations

The following abbreviations are used in this manuscript:

ICG	Indocyanine green
NPs	Nanoparticles
ROS	Reactive oxygen species
DNA	Deoxyribonucleic acid
PLGA	Poly(lactic-co-glycolic acid)
SiNPs	Silicon nanoparticles
HSA	Human serum albumin
-SH	Mercapto groups CSMS
APTES	(3-aminopropyl)triethoxysilane
MPTS	(3-mercaptopropyl)trimethoxysilane
CSMS	Mesoporous silica microspheres
TMOS	Tetramethoxysilane
GO	Graphene oxide
US	Ultrasonic treatment
